# Laser Analgesia Associated with Restorative Dental Care: A Systematic Review of the Rationale, Techniques, and Energy Dose Considerations

**DOI:** 10.3390/dj8040128

**Published:** 2020-11-12

**Authors:** Riccardo Poli, Steven Parker, Eugenia Anagnostaki, Valina Mylona, Edward Lynch, Martin Grootveld

**Affiliations:** 1 Leicester School of Pharmacy, De Montfort University, Gateway House, Leicester LE1 9BH, UK; steven.parker@my365.dmu.ac.uk (S.P.); eugenia.anagnostakis@my365.dmu.ac.uk (E.A.); vasiliki.mylona@my365.dmu.ac.uk (V.M.); edward.lynch@hotmail.com (E.L.); mgrootveld@dmu.ac.uk (M.G.); 2 School of Dental Medicine, University of Nevada Las Vegas, Las Vegas, NV 89154, USA

**Keywords:** dentistry, laser, analgesia, restorative, pain

## Abstract

It is a common experience amongst laser dentists and patients that mid-IR wavelength application in cavity preparation may be achieved without causing any associated pain. The erbium family of lasers (Er,Cr:YSGG 2780 nm and Er:YAG 2940 nm) are frequently used without employing injectable local anesthesia as an adjunct: the phenomenon arising from the application of these devices is known as laser analgesia. This review seeks to apply a systematic approach to the examination of appropriate published studies but also to highlight the need for much more structured clinical investigations that consolidate photonic dose and methodology. A search of published data using PRISMA criteria was carried out to examine clinical trials into laser analgesia in conjunction with restorative dentistry, applying inclusion and exclusion criteria. From this, 10 published articles were selected for analysis. Suitability assessment was carried out, using a modified Cochrane risk of bias methodology. In 8/10 of the included studies, laser-induced analgesia is claimed to be better and effective, while in 2/10 of the studies, no difference was exhibited compared to the control group. Statistical analysis of three split mouth studies concluded that only one of these investigations reviewed demonstrated a significant analgesic effect for laser treatment while the other two did not support this observation. From this data, it is inconclusive to assess the predictability of laser analgesia in cavity preparation. A possible rationale and laser operating parametry has been discussed. Successful implementation of this treatment modality remains technique sensitive and subject to further investigation.

## 1. Introduction

During the development of laser applications in dentistry, several wavelengths have been used in an attempt to perform caries removal and cavity preparation. However, the first devices (i.e., CO_2_ 10,600 nm and Nd:YAG 1064 nm) had insufficient ability to penetrate into dental hard tissues and produced major thermal side-effects, such as melting, carbonisation, cracks in the surrounding dental tissues, and increases in pulpal temperature [1,2,3,4]. However, the introduction of erbium family lasers (Er,Cr:YSGG 2780 nm and Er:YAG 2940 nm) has now allowed a more effective removal of oral hard tissues (enamel, dentine, cementum, and bone) and this major benefit is coupled with a low risk of thermal damage.

The use of erbium lasers has been reported to be a more comfortable method of cavity preparation than a bur, with a significantly reduced requirement for local anaesthesia [5,6,7,8]. Cozean et al. demonstrated that the Er:YAG wavelength can be efficiently used for painless caries removal and cavity preparation, and that less than 2% of the patients involved required local anaesthesia [9]. Hibst et al. [6] found that only 6% of 103 patients (206 cavities) required injected local anaesthesia and the vast majority rated the laser preparation as “more comfortable” when compared to bur tissue removal.

During the last two decades and in order to address and suppress the potential for pain associated with dental treatment, every dental practitioner has been using injectable local anaesthesia; in itself, such adjunctive treatment might represent a further source of anxiety and dental fear, with the ability to trigger further phobic reactions. Possible alternatives that have been attempted or used to bypass this anxiety have been hypnotic analgesia and conscious sedation with nitrous oxide, although it is not possible to suppress pain perception up to a point where the patient may experience a painless restorative procedure. Topical anaesthetics and electrically induced anaesthesia have also been tested in several investigations, albeit with contrasting results. The use of intra-venous sedation or general anaesthesia in restorative dentistry is considered to be reserved for patients who are completely uncooperative; thus, it is not usually included amongst these alternatives [10,11,12,13,14].

Laser analgesia is a non-invasive, non-destructive, and non-thermal bio-modulating technique with the ability to reduce or suppress painful sensations; it is obtainable in a low-energy-level irradiation form. The causes of this effect are not completely clear, even though several theories have been proposed. Possible explanations are represented by the photo-acoustic effect and its actions within the gate control pathway, direct and indirect influences of laser energy on nerves and nociceptors, modifications of the Na+-K+ pump systems, and the bio-resonance and biochemical modifications that laser energy is able to induce. Techniques are available that result in a laser-induced analgesic effect, and methods for computing the energy dose. Notwithstanding, it is not a profound anaesthesia since it is unable to suppress all sensations. The aim of this laser application is the suppression of nerve and nociceptor activities, processes creating a lack of pain perception and diminishing dental pulp and soft tissue reactivity [15,16,17].

It is appropriate to record that laser–tissue interaction has a potential for thermal rise, through the very nature of photothermal energy transfer; it is necessary for the clinician to apply precise laser and air/water co-axial operating parameters in order to avoid any unwanted dental and pulpal damage, both during the induction of laser analgesia and consequent cavity preparation [18,19].

Several wavelengths have been investigated in order to obtain laser analgesia: visible (445–670 nm), diodes (810–980 nm), Nd:YAG (1064 nm), erbium family lasers (Er,Cr:YSGG 2780 nm and Er:YAG 2940 nm), and CO_2_ (10,600 nm).

Reflecting elevations in fear and perceived fear in relation to dental care, patients are therefore prone to seek alternative therapeutic experiences and many of them prefer to avoid traditional restorative treatments, since there is a strong association between their fear level and the use of the traditional turbine and bur, along with its associated noise, smell, and vibrations [20,21,22,23].

According to previous reports, in relation to receiving restorative dental treatment, the percentage of anxious subjects appears to be on the increase. Data relating to US and European investigation can be helpful in providing some context to the prevalence of odontophobia; in 1983, it was estimated to be in the range of 10–12% [21]; later, in young (child and adolescent) patients, it was determined to range from 6% to as much as 52% in 1992 [21]; and finally, it attained a figure of about 50% in 2009 [22].

The same trend appears to occur amongst odontophobic patients; this group represents the most unwilling to cooperate and difficult to treat subjects, estimated to be 3–5% in 1983 [21], up to the most recent data available (2004) of 4–16% of adults, and 7–20% of children [23]. Indeed, we may consider that there is an increasing trend of anxiety and phobia related to dental treatments.

There is considerable dichotomy between the predominately anecdotal and patient-centred approach to laser analgesia, compared to the constraints of rigorous randomised clinical trials and therein lies a good deal of the challenge to establish a basis for investigation or to successfully examine published data. The introduction of the first laser within dentistry in 1989 (American Dental Laser DLase 300 Nd:YAG 1064 nm) was heralded by considerable promotion, albeit based upon anecdote and unsubstantiated claims. Early published reports [24,25,26,27,28] were evidently supported by the manufacturer and unfortunately, many of these early studies formed part of the product promotion. A significant randomized trial in 1995 [29] was unsuccessful in determining predictable and statistically significant advantages of laser analgesia as adjunct to tooth cavity preparation, despite recording patient acceptance of the technique over needle local anaesthesia.

In order to seek further clarity, a systematic review was carried out to examine the effectiveness of laser-induced analgesia in restorative dentistry. As an example, one approach in treating patients requiring restorative dentistry is detailed as an example of the approach, and is detailed in the discussion.

## 2. Materials and Methods

### 2.1. Search Strategy

An electronic search was conducted relating to laser analgesia applications in all fields of dentistry from 17 July to 19 July 2020. Databases used were PubMed, Cochrane, and Google Scholar with the following MeSH terms, keywords, and their combinations: Laser AND (analgesia OR pain) AND (caries OR cavity preparation).

Duplicate articles were excluded and the initial number of 103 was reduced to 79.

After applying the additional filters (published within the last 15 years, only clinical trials in humans, and only English language reports), the preliminary number of 79 articles was reduced to 16.

Titles and abstracts of the above articles were independently screened by two reviewers via application of the following criteria. In case of any disagreements arising, these were satisfactorily resolved by discussions.

Inclusion criteria:

- Laser used;
- At least 10 patients per group;
- Only clinical trials; and
- Laser use applicable to dental procedure to be carried out.

Exclusion criteria:

- Duplicates or studies with the same ethical approval number;
- Pain control studies where non-laser analgesia was employed;
- Less than 10 patients per group; and
- No clinical trials or study protocols or pilot studies.

After screening and implementation of the eligibility criteria, a total of 9 articles were retained.

**Figure 1:**
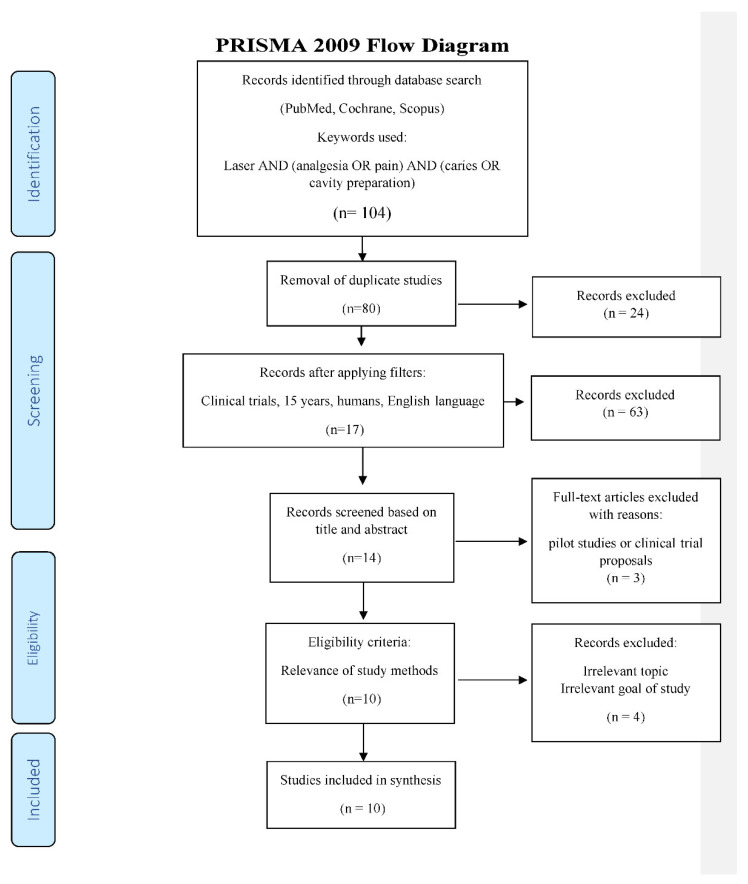
PRISMA flow-chart of selected criteria for the included article reports.

In accordance with the PRISMA statement [30], details of the selection criteria are presented in Figure 1.

### 2.2. Data Extraction

Having reached a consensus regarding the selection of included articles, the four reviewers involved subsequently extracted data regarding:

- Citation (first author and publication year);
- Type of study/number of patients;
- Test/control group;
- Aim/approach;
- Laser parameters applied; and
- Outcome.

### 2.3. Quality Assessment

Subsequent to data extraction, articles were further evaluated by assessing their risk of bias assessment. The Cochrane risk of bias tool [31] was modified according to the requirements of this systematic review.

The risk of bias was determined according to the number of “yes” or “no” responses to the parameters provided below, which were allocated to each study:

- Randomization;
- Sample size calculation and required number included;
- Baseline situation similar;
- Blinding;
- Parameters of laser use described appropriately and calculations correct;
- Power meter used;
- Numerical results available (statistics);
- Outcome data complete; and
- Correct interpretation of data.

The classification was performed according to the total number of “yes” answers to the above questions. For the current study, the degree of bias was computed according to the score limits provided below:

High risk: 0–3;
Moderate risk: 4–6; and
Low risk: 7–9.

## 3. Results

### 3.1. Primary Outcome

The primary goal of this review was to explore the effectiveness of laser-induced analgesia in clinical dental practice.

### 3.2. Data Presentation

The results of the included studies are presented in Table 1.

### 3.3. Quality Assessment Presentation

The risk of bias evaluation is presented in Table 2.

In total, 3/10 articles showed a low risk of bias:

One article [10] scoring 8/9; and
Two articles [32,33] scoring 7/9.

In total, 4/10 articles showed a medium risk of bias:

Three articles [35,36,39] scoring 5/9; and
One article [34] scoring 4/9.

In total, 3/10 articles showed a high risk of bias:

Three articles [37,38,40] scoring 3/10.

It can be seen that only four articles [10,33,35] had sufficient and reproducible description of the laser protocol applied, while only in one study [10] was a power meter used, and in two others [10,32], sample size calculation was done before the clinical trial and subsequently the required number of patients was included.

### 3.4. Analysis of Data

According to the type of study, the included articles can be divided into two categories and subsequently critically appraised regarding the primary outcome:

- In split-mouth RCTs, 3/5 [33,36,39] studies showed that the laser group achieved statistically significant better results compared to the control group while 2/5 [32,35] studies showed no difference between these groups.
- In the parallel RCT study [35], results showed a significant difference in pain perception regarding the laser group.
- In clinical trials, the range of positive treatment outcomes was between 66.7% and 89.5% in the remaining five studies [10,34,37,38,40].

Statistical analysis was performed on a combination of three split-mouth studies in which it was possible to classify the responses as pain experience or no pain, and these data are provided in Table 3. Firstly, the Cochran–Mantel–Haenszel (CMH) test was applied to the complete dataset in order to determine if the relative proportions of pain or no pain experienced following the laser treatment and corresponding control groups are consistent or not within all 3 sets of 2 × 2 contingency tables. Hence, the null hypothesis was that there is a consistent difference in these proportions across all three studies explored here. However, the CMH test revealed a high level of inconsistency between the results acquired in these studies (*p* = 6.04 × 10^–5^).

Secondary 2 × 2 contingency table analysis of each study dataset independently showed that for the Sarnadi [32] and Chan [10] studies, there was no significant association between the proportions of participants experiencing pain above a selected threshold and the treatments applied (laser vs. control), χ^2^ test (with Yates’ correction for continuity) *p* values of 0.39 and 0.51 (ns), respectively. However, for the Liu study, this *p* value was 2.36 × 10^−6^, and data therein clearly demonstrated that the laser system applied exerted a major analgesic effect, which was very highly statistically significant. Therefore, it may be concluded that only one of these investigations reviewed demonstrated a significant analgesic effect for laser treatment, but two other corresponding studies performed did not support this observation.

## 4. Discussion

The systematic review offers some insight to the approach when employing laser-assisted analgesic techniques. Considering the impact of the object of the technique among a large potential population of patients, there is some regret that the quantity and specificity of objective investigations remains small and not without some risk of bias. It is difficult to understand the lack of quality investigations into technique, operating parameters, and predictability relative to patient acceptance, when it is beyond question that the impact on delivering painless dentistry remains an important aspect of gaining patient confidence.

The search criteria in this literature review allowed some limited consistency in results to enable possible techniques and laser operating parameters to be explored. It is safe to reflect on the wide range of techniques and laser dose in terms of energy per pulse, frequency, and length of application time.

From the studies examined, the ones that offered detail in terms of technique and laser irradiance values have either adopted a dedicated pre-operative analgesia stage [33,35,36,37,38] or utilised effective but tolerable laser ablation power and draw comparisons with a similar treatment using a rotary bur [10,32,38,39,40]. Two of the studies analysed utilised NIR laser wavelengths to achieve laser analgesia alone [33,38]. Of the Erbium family, five studies used Er:YAG 2940 nm [10,32,38,39,40], two used the Er,Cr:YSGG 2780 nm wavelength [34,40], and one study used both wavelengths [37].

Of those studies with statistical analysis, five [10,32,33,36,39] offered a statistically significant benefit of laser use over alternative modalities, whereas another was equivocal in outcome with no statistical significance [35]. In the Poli et al. study [34], utilising both methods, it was discovered that amongst 30 patients exposed to laser-assisted restorative dentistry, 80% perceived no pain, and none required local anaesthesia; the use of pain scales to assess laser benefit provided a general level of acceptance over conventional treatment alternatives. Although free running pulsed (FRP) lasers exert significant effects on pain, researchers have yet to reach a definitive conclusion over the mechanisms of such effects.

Only recently has a protocol focused on achieving a predictable onset of analgesia using an erbium family laser system been proposed [34,41].

**Figure 2:**
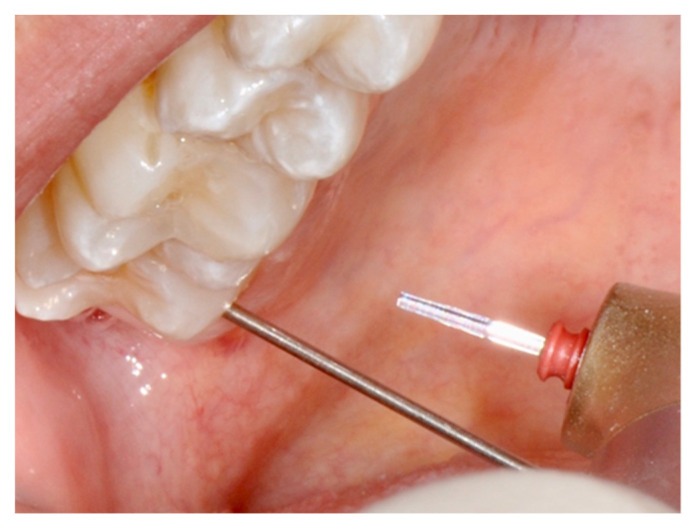
Example of tip kept at 10 mm for the rabbit technique of laser-induced analgesia. Irradiation at tooth neck level.

**Figure 3:**
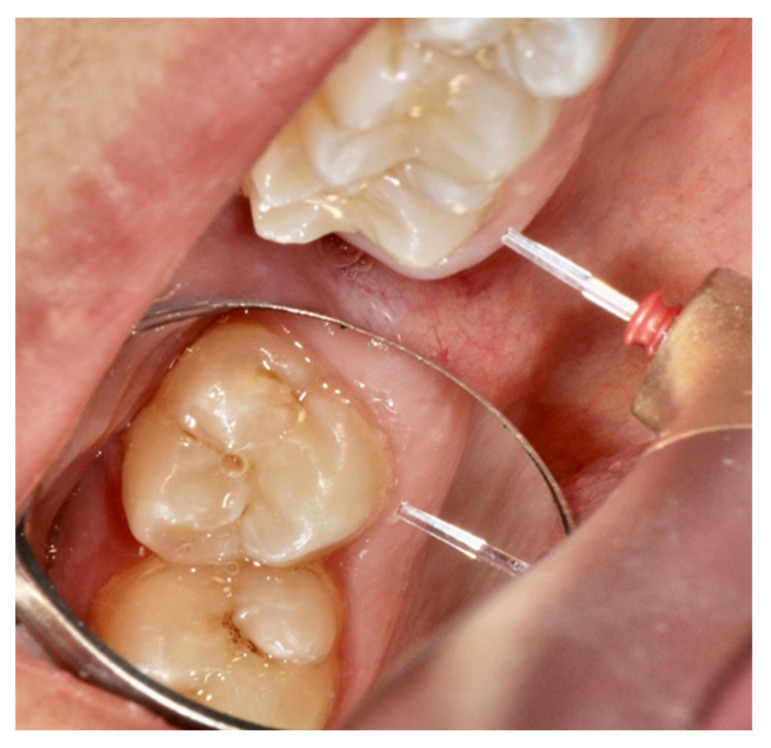
Example of the tip kept at 1 mm of distance from the tooth neck as in the turtle technique.

**Figure 4:**
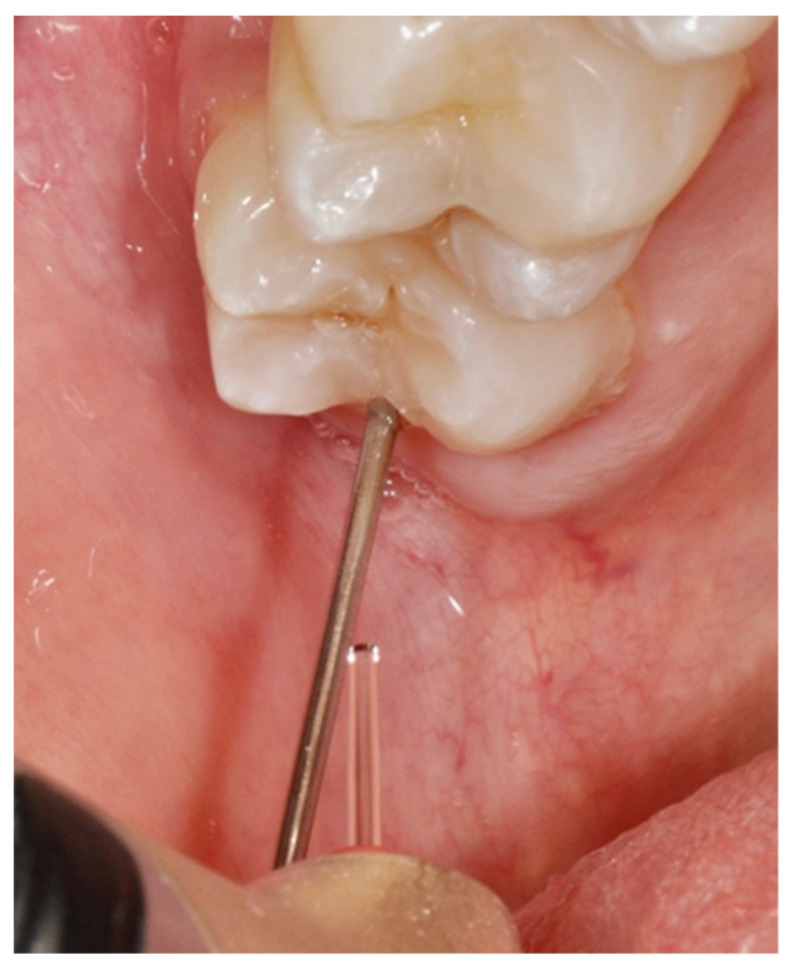
Rabbit technique: irradiation from the occlusal side of the tooth.

Two specific techniques have been described in order to attain a reliable level of analgesia in restorative dentistry [34,41]. The “rabbit” technique, also known as the “hare” technique, assumes the use of high energy settings at the beginning of treatment. Key to any success with either technique is the specificity of applied fluence and to achieve this, and one possible approach is the use of a “spacer” to preserve the exact “tip to tissue” distance, as illustrated in Figure 2, Figure 3 and Figure 4. The parameters set are then immediately able to create a tooth cavity, but initially, the handpiece is kept at a distance of 6–10 mm from the target, in order to obtain a de-focused beam and a low fluence that is not perceived as pain (Figure 2 and Figure 3).

The tip is moved all around the tooth neck, and following several tens of seconds (up to two minutes in total), the tip is placed closer to the tooth surface, and when it is at 0.5–1 mm from that site, the ablation commences. If the patient then feels any discomfort, the handpiece can be moved aside and the analgesia phase starts again. When the dentine is penetrated, the parameters are lowered, or the tip is placed further away to reduce energy density.

The second technique is known as the “turtle” (or “tortoise”) strategy. Differently from the previous approach, at first, the energy and power settings are low to create a low level of irradiation. The tip is then placed at an ablative distance (0.5–1 mm) from the surface, but the formation of the cavity does not occur until the energy settings increase and overcome the ablation threshold of the specific tissue targeted. When the dentin is reached, the parameters are then modified accordingly (Figure 4).

With both techniques, primarily, the irradiation process is always considered a low-level laser therapy (LLLT). With the rabbit technique, however, the risk to have a greater sensitivity is higher, since the energy is immediately set at ablative values and hence, if the distance is reduced, the impact of the beam may be perceived by the patient. On the contrary, with the “turtle” technique, it is easier to obtain the laser analgesia, and the risk of discomfort is generally lower [41]. It is also possible to combine the two different techniques to take advantage of both and also increase the likelihood of attaining a painless restorative treatment [34].

The settings necessary to induce laser analgesia have already been investigated through published studies [34,41]; however, further research is required in order to determine the exact amount of medium-IR energy, and the correct parameters necessary to produce a more predictable protocol and a reliable laser analgesia-based treatment regimen. In the absence of more data, it might be appropriate to consider the general dose range in the treatment of dento-alveolar pain associated with tissue injury and orthodontic pain. In this way, according to Bjordal et al. [42], the optimal dose on acute pain in the first 72 h following the injury should be 7.5 J/cm^2^. Other authors proposed greater levels of energy, such as Lizarelli [43], who indicated a dose within the range 5–20 J/cm^2^ for severe pain. A fluence of 35 J/cm^2^ was suggested by another group to reduce orthodontic pain, since a level of only 5 J/cm^2^ was not found to be effective [43]. In the current available literature, similar protocols reported conflicting results [17,44].

In examining possible mechanisms that may contribute to the positive patient experience during laser therapy, these are most likely additive in nature rather than being mutually exclusive.

According to the bio-psycho-social model of pain [45], many different aspects can influence pain perception. Environment, pain behaviour, psychological distress, attitudes and beliefs, pain modulation, health conditions, nerve and nociceptor activity, and/or neuropathy are all possible elements that can interact with each other. Therefore, it seems likely that lasers have different effects on one or many of these factors [46]. The limited pulp temperature increase [47,48,49] appears to represent a further element that limits the pain experienced, both during and after the applied treatment.

FRP laser systems reduce the annoyance factor, which is a combination of the pressure applied to the tooth, the vibrations and noise recorded through the bones of the skull, and the heat and smell generated at the interface between the tooth and cutting instrument.

Numerous host response elements seem to be combined and involved in modulation and suppression of nociception. All of them are able to reduce, modify, or block nerve impulse transmission and conduction from several nociceptors. These morphological and physiological neuronal changes are, however, reversible after photobiomodulation [50]. Points of consideration may be summarised as follows:

- The gate control pathway [51,52];
- Indirect influence on the nervous system [53,54,55,56,57];
- Direct inhibition of the nervous system [15,16,35,58,59,60];
- Effects of laser energy on oral environment biochemistry [17,61,62,63,64,65,66,67,68,69,70,71,72,73];
- Photo-acoustic effect of pulsed lasers [46];
- Techniques to induce laser analgesia [34,41]; and
- Energy dose considerations [17,34,41,43,44,61].

## 5. Conclusions

From this review, it can be pointed out that laser-induced analgesia can be considered a possibly effective alternative treatment modality in affecting pain perception. An important role is also attributed to the altered patient responses due to the limited invasiveness of laser treatments and consequently to the minor level of anxiety.

Although none of the studies declared any adverse effect, the lack of consistent laser operating parameters and inconsistent statistical significane regarding outcomes over conventional analgesic and anaesthetic techniques does not allow firm conclusions to be formed as to which laser wavelength is superior or what parameters might be employed to achieve predictability. The inconsistencies of non-standardised study design, varying parametry or lack of essential laser operating values, and lack of statistical analysis together have failed to underline a strong protocol of how the mid-IR wavelengths may be applied in achieving laser analgesia.

Above all, laser analgesia remains a profoundly technique-sensitive modality to achieve success. As such, this application can be considered safe as long as the correct parameters are applied, although it is clear that more clinical studies with standardised protocol should be performed in order to deeply explore and understand this procedure.

## Figures and Tables

**Table 1:** Data evaluation of the included studies.

Citation [Ref]	Type of Study/Number of Patients	Test/Control Group	Aim/Approach	Laser Parameters	Outcome
Sarmadi et al. (2018) [32]	Split-mouth RCT/25 patients with at least 2 primary caries of equal size and same location (occlusal or interproximal)	2940 nm (28 cavities)/rotary bur (28 cavities)	Discomfort and Pain (VAS)	Enamel preparation: 250–300 mJ, VSP pulse, 30 Hz, water/air Dentin preparation: 200–300 mJ, VSP/SP pulse, 10–20 Hz, water/air Excavation dentin caries: 200–300 mJ, VSP/SP pulse, 20–30 Hz, water/air Excavation deep caries: 150–250 mJ, SP pulse, 5–15 Hz, water/air	Laser group: 10/28 patients required LA Bur group: 15/28 patients required LA No difference immediately after tx (*p* = 0.881)
Liang et al. (2016) [33]	Split-mouth RCT/30 patients, healthy maxillary first premolars examined (one tooth/group)	PBM with 904 nm (30 teeth)/placebo effect (30 teeth)	Pulpal response (EPT) 2 min prior and immediately after irradiation	PBM: Average Power 30 mW, 25 Hz, 50% duty cycle, buccal surface for 60 s, 3.6 J, 1 cm^2^ spot size	Significant difference *p* < 0.0001
Poli et al. (2015) [34]	Clinical trial/30 patients with a single cavity	2780 nm	Pulpal response (EPT) for pre-, intra- and post-treatment evaluation Pain (VAS)	ANALGESIA: The delivery tip used was the MGG6 sapphire tip, diam- eter 0.600 mm, length, 6 mm (beam spot area = 0.283 mm2). Power of 0.1 W and then 0.2 W (energy per pulse 10 and 20 mJ), for 30 s each (without air/water spray), 10 Hz, tip at 10 mm from the tooth using a spacer. Subsequently, the power was increased to 0.5 and then to 1 W (33 and 67 mJ) for 60 s each, with a spray of 15% water (*10 mL/min) and 20% air, 15 Hz, keeping the same distance. Hard tissues were preconditioned with 2 W for 30 s, spray 50% water (*20 mL/min) and 80% air, 15 Hz, tip at 1 mm from the tooth. The laser was defocused if the patient felt discomfort	VAS: 0–1 values at 24/30 (80%) of patients EPT values variations: No difference (*p* > 0.05)
Chan et al. (2012) [10]	Split-mouth RCT/44 patients having a cavity by bur at bilateral premolars (one tooth/group)	Nd:YAG + sham EMLA (44 teeth)/EMLA + sham laser (44 teeth)	Pain (EPT and VAS)	ANALGESIA: 150 μsec, AP 1.1 W, 15 Hz; 60–87 mJ, 0.3–0.45 W/cm^2^, 73–107 J/cm^2^, total energy, 211- 312 J, 320 μm fiber, scanning motion (speed at 3 mm/s) approximately 1 mm (spot diameter = 6 mm) from the buccal and lingual/palatal cervical areas for 240 s	Both groups achieved analgesic effects No significant difference in EPT and VAS scores between groups
Belcheva et al. (2014) [35]	Parallel-group RCT/90 patients. 1 or more dentine carious lesions without pulp involvement or pain. Occlusal or proximal surface of a primary or a permanent molar	Er:YAG (45 patients)/Rotary bur (45 patients)	Pain (universal pain assessment tool)	200–300 mJ/20 Hz, water 8 for the permanent teeth. 100–200 mJ/ 20 Hz, water 8 for the primary teeth.	Laser group significant lower pain (*p* = 0.005) Laser group significant difference in low degree of pain (*p* < 0.005) and in severe degree (*p* < 0.01) compared to control group, respectively
Tanboga et al. (2012) [36]	Split-mouth RCT/10 patients (6–9 y.o) with primary molars (one tooth/group)	LLLT (Er:YAG) + Er:YAG prep (10 teeth)/Er:YAG prep (10 teeth)	Pain (VAS)	ANALGESIA: at a distance of 2 mm from the tooth surface on the gingival margin and slowly moved for 2 min, 60 mJ, 20 Hz, 250 ms pulse, additional water spray	Test group significant better *p* = 0.004
Genovese et al. (2008) [37]	Clinical trial/50 patients (6–12 y.o) required both hard and soft tissue therapy, without anaesthesia	Er:YAG and Er,Cr:YSGG Cavity prep: 20 permanent molars and 30 deciduous molars soft tissues: 23 frenectomies, 12 gingivectomies, 15 operculectomies	Patient’s experience (Wong-Baker modified facial image scale)	ANALGESIA: 0.5–2.5 W, 20 Hz, 75 mJ, Air 20%, Water 15%, at a distance of 3 mm from the tooth surface, in defocused mode on the gingival margin (1–3 mm), and slowly moved for 2 min	Pain values 1–2 at 44/50 (88%) of patients tested for cavity preparation
Matsumoto et al. (2007) [38]	Clinical trial/45 patients (95 teeth) with primary carious lesions in vital teeth	Cavity preparation in enamel and dentin with Er:YAG (95 teeth)	Pain (4-point scale)	CAVITY PREP: 100–700 mJ, 80–700 ms pulse, 8–20 Hz, water 12 mL/min	Pain values 1–2 at 85/95 (89.5%) of patients
Liu et al. (2006) [39]	Split-mouth RCT/40 patients (4–12 y.o) with two maxillary anterior carious teeth, same type of lesion and approximately equal-sized cavities	Er:YAG (40 teeth)/rotary bur (40 teeth)	Pain (simple modified face scale)	CAVITY PREP: 700 mJ, 10 Hz, non-contact 1 mm distance, 800 μm tip, water 24 mL/min	Laser group significantly better than control *p* < 0.001
Boj et al. (2005) [40]	Clinical trial/33 patients (8–16 y.o.) required restorations in permanent teeth	Er,Cr:YSGG used for restorations in permanent teeth	Pain (Wong-Baker facial image scale)	Manufacturer’s recommendations	Pain values 1–2 at 22/33 (66.7%) of patients

**Table 2:** Risk of bias evaluation of the included studies.

Citation [Ref]	Randomization	Sample Size Calculation and Required Number Included	Baseline Situation Similar	Blinding	Parameters of Laser Use Described Appropriately and Calculations Correct	Power Meter Used	Numerical Results Available (Statistics)	No Missing Outcome Data	Correct Interpretation of Data	Total Score/ 9
Sarmadi et al. (2018) [32]	yes	yes	yes	yes	no	no	yes	yes	yes	7
Liang et al. (2016) [33]	yes	no	yes	yes	yes	no	yes	yes	yes	7
Poli et al. (2015) [34]	no	no	no	no	yes	no	yes	yes	yes	4
Chan et al. (2012) [10]	yes	no	yes	yes	yes	yes	yes	yes	yes	8
Belcheva et al. (2014) [35]	yes	no	yes	no	no	no	yes	yes	yes	5
Tanboga et al. (2012) [36]	yes	no	yes	no	no	no	yes	yes	yes	5
Genovese et al. (2008) [37]	no	no	no	no	no	no	yes	yes	yes	3
Matsumoto et al. (2007) [38]	no	no	no	no	no	no	yes	yes	yes	3
Liu et al. (2006) [39]	yes	no	yes	no	no	no	yes	yes	yes	5
Boj et al. (2005) [40]	no	no	no	no	no	no	yes	yes	yes	3

**Table 3:** Statistical analysis was performed on a combination of three split-mouth studies in which it was possible to classify the responses as pain experience or no pain. Data extracts allowed comparative patient numbers to be computed and assessed.

Study	Pts Total/	Cavities by Group	Treatment	Response	
				Pain	No Pain
Sarnadi [32]	1	25/28	Control	13	12
		25/28	Laser	9	16
Chan [10]	2	44/44	Control	18	26
		44/44	Laser	14	30
Liu [39]	3	40/40	Control	29	11
		40/40	Laser	7	33
